# Probiotics as Adjunctive Treatment for Patients Contracted COVID-19: Current Understanding and Future Needs

**DOI:** 10.3389/fnut.2021.669808

**Published:** 2021-06-10

**Authors:** Jiangying Peng, Meng Zhang, Guoqiang Yao, Lai-Yu Kwok, Wenyi Zhang

**Affiliations:** ^1^Key Laboratory of Dairy Biotechnology and Engineering, Ministry of Education, Inner Mongolia Agricultural University, Hohhot, China; ^2^Key Laboratory of Dairy Products Processing, Ministry of Agriculture and Rural Affairs, Inner Mongolia Agricultural University, Hohhot, China

**Keywords:** COVID-19, SARS-CoV-2, acute respiratory disease, pneumonia, probiotics, adjunctive treatment, gut-lung axis

## Abstract

The coronavirus disease 2019 (COVID-19) is caused by a novel coronavirus, severe acute respiratory syndrome coronavirus 2 (SARS-CoV-2), which rages all over the world and seriously threatens human life and health. Currently, there is no optimal treatment for COVID-19, and emerging evidence found that COVID-19 infection results in gut microbiota dysbiosis. The intestinal microbial richness of patients of COVID-19 does not return to normal levels even six months after recovery, but probiotic adjunctive treatment has been found to restore gut homeostasis. An updated PubMed search returned four finished clinical trials that supported the use of probiotics as adjunctive treatment for COVID-19, while at least six clinical trials aiming to investigate beneficial effects of probiotic intake in managing COVID-19 are currently in progress worldwide. Here in we tentatively summarized the understanding of the actions and potential mechanisms of probiotics in the management of COVID-19. We also highlighted some future needs for probiotic researchers in the field. The success in using probiotics as adjunctive treatment for COVID-19 has expanded the scope of application of probiotics, meanwhile deepening our knowledge in the physiological function of probiotics in modulating the gut-lung axis.

## Introduction

The coronavirus disease 2019 (COVID-19) caused by a novel coronavirus, severe acute respiratory syndrome coronavirus 2 (SARS-CoV-2), has been raged across the globe since the end of 2019, seriously threatening human life and health (1, 2). Yet, the number of COVID-19 cases is still growing around the world. The severity of COVID-19 has brought and will certainly continue to bring tremendous financial and medical burdens to the society; thus, cost-effective health management strategies are urgently needed.

The symptoms of COVID-19 include fever, dyspnea, dry cough, upper and/or lower respiratory tract symptoms, myalgia, fatigue, diarrhea, or a combination of these 3–6 days after exposure (3–5). Other symptoms include headache, sore throat, rhinorrhea, and gastrointestinal disorders (4, 6). At the beginning of the outbreak, several drugs have been applied to manage the disease, including remdesivir and favipiravir. Among them, remdesivir was found to be the most promising drug that effectively cured the first COVID-19 patient in the United States (7). Currently, multiple effective vaccines have been developed and are available to people worldwide. However, the decrease in gut microbiota richness and dysbiosis in patients with COVID-19 did not return to normal levels even six months after recovery, and the persistent gut dysbiosis was linked with the severity of disease and inflammatory reaction (8). Previous studies have shown that microbial diversity is a key determinant of microbial ecosystem stability (9, 10). A stable intestinal ecosystem enhances colonization resistance against opportunistic pathogens, while malnutrition hinders the recovery of intestinal health and lung function of patients with COVID-19 (11–13). Therefore, targeted auxiliary microecological therapy that helps restore healthy gut microbial communities is anticipated to accelerate full recovery from COVID-19 (8).

Probiotics are live microbes that, when administered in adequate amounts, confer health benefits on their hosts (14). To date, four clinical studies around the world have successfully applied probiotics as adjunctive treatment in managing COVID-19 (5, 15–17), while there are more than six undergoing clinical trials (18–20). The present review summarized the understanding of the actions and potential mechanisms of probiotics in the management of COVID-19, and highlighted some future needs for probiotic researchers in the field.

## Probiotics as Adjunctive Treatment for Patients Contracted COVID-19

In response to the outbreak of COVID-19, a Chinese infectious disease scientist proposed a treatment plan called “four-antibody, two-balance,” which was successfully implemented for treating SARS-CoV-2-associated acute respiratory distress syndrome (16). The four antibodies represented antiviral, antishock, antihypertension, and anti-infection therapy, while the “two-balance” referred to acid-base balance of water electrolysis and microecological balance (16). The microecological balance was considered to be of particular importance in the treatment, as microecological imbalance would lead to secondary infection of bacteria after recovering from the viral infection (16).

An updated PubMed search recovered four finished clinical studies from different countries that found positive effects of applying probiotics as adjunctive treatment in COVID-19. For example, Wu et al. found that taking a large dose of probiotics as adjuvant treatment significantly improved the symptoms of the disease, accompanied by the reduction in inflammation and recovery from gut microbiota abnormalities (17). A case study report found that some COVID-19 patients suffered from microbial dysbiosis characterized by decreased levels of intestinal *Lactobacillus* (*L*.) and *Bifidobacterium* (*B*.) (16). Xu et al. observed that most COVID-19 patients with relatively mild symptoms had received probiotics (5). Baud et al. summarized data from a large body of human studies and listed different probiotics that might have relevance to lowering burden brought by COVID-19, including *Lactobacillus casei, Lactobacillus plantarum, Lactobacillus rhamnosus, Lactobacillus gasseri, Bifidobacterium bifidum, Bifidobacterium longum, Bifidobacterium breve, Leuconostoc mesenteroides*, and *Pediococcus pentosaceus* (15). Currently, there are at least six ongoing clinical trials aiming to investigate the beneficial effects of applying probiotics as adjunctive treatment in managing COVID-19 (18–20). It is noteworthy that a research scientist from Rutgers University designed a microbiota formula for early management of COVID-19 (20). This formula is a new prescription targeting to regulate the gut microbiota, aiming to prevent severe complications and hospitalization in high-risk patients of COVID-19 who suffer from underlying conditions like obesity or type-2 diabetes (20). The US Food and Drug Administration has approved the new formula for new drug clinical trial in a record time (20). Although this formula is different from ordinary probiotics-based products, the novel ecological approach focusing on managing the gut microbiota seems promising.

## The Understanding of The Actions and Potential Mechanisms of Probiotics in the Management of COVID-19

The study of human microflora and the function of probiotics can be traced back to the longevity theory proposed by Élie Metchnikoff, a famous Russian scientist who won the Nobel Prize in 1908. Over the last decades, the advent of high-throughput sequencing technologies has revealed much information of the mechanisms behind the health-promoting effects of probiotics in maintaining gut homeostasis. A number of random clinical trials (RCTs) were performed to evaluate the beneficial role of probiotics in adjunctive treatment of various gastrointestinal diseases, such as inflammatory bowel disease, irritable bowel syndrome, and antibiotic-associated diarrhea (21). Direct and indirect evidence of health outcome data generated from various clinical trials suggested that effective probiotic adjunctive treatments rely largely on modulating the gut and lungs, and the gut-lung axis.

### Restoration of a Healthy Gut Microbiota

Probiotics may enhance restoration of a stable gut microbiota in the colonic environment and prevent colonization by pathogenic organisms via a number of mechanisms (22, 23). For example, probiotics can bind to gut epithelial cells and result in competitive inhibition against gut wall adhesion of pathogenic microorganisms (22, 24). Once adhered to the gut epithelial cells, probiotics release substances that prevent the growth of pathogens and modulate intestinal permeability (22). In addition, probiotics can restore the intestinal health by attenuating inflammation and strengthening the epithelial barrier (25, 26). Firstly, probiotics can enhance the intestinal barrier function and prevent the invasion of pathogenic microorganisms by increasing the secretion of mucin (25, 27). Mucin is a highly glycosylated substance distributing in the cell membrane, and it is secreted into the intestinal cavity to form a mucus layer. The mucus layer is the first line of defense responsible for ensuring the integrity of the intestinal barrier, and pathogenic microorganisms would not be able to reach the gut epithelial cells without breaking through the mucus layer. Secondly, probiotics may prevent the growth of pathogens directly by secreting antibacterial factors, such as antibacterial peptides, defensins, short-chain fatty acids, and bacteriocins (28, 29). Thirdly, probiotics may enhance the intestinal epithelium barrier function by increasing the expression of tight junction proteins (25).

Scarce clinical data are hitherto available; however, deprivation of important gut commensals, like *Lactobacillus* and *Bifidobacterium*, has been consistently reported in some patients of COVID-19 (16). These gut microbes are known to play a significant role in maintaining colonic homeostasis. Similarly, a six-month follow-up study found that the gut microbiota richness was compromised in COVID-19 patients compared with healthy individuals (8). Higher abundances of opportunistic pathogens, including *Collinsella aerofaciens, Collinsella tanakaei, Streptococcus infantis*, and *Morganella morganii*, accompanied by an elevated functional capacity for nucleotide de novo biosynthesis, amino acid biosynthesis and glycolysis, were observed in the fecal metagenomes of high SARS-CoV-2 infectivity patients, whilst the fecal metagenomes of low-to-none SARS-CoV-2 infectivity patients were characterized by abundant short-chain fatty acid producing-bacteria, such as *Parabacteroides merdae, Bacteroides stercoris, Alistipes onderdonkii*, and *Lachnospiraceae bacterium* (30). Thus, one important goal of the probiotic adjunctive treatment in managing COVID-19 would be to restore the gut microbiota diversity, composition, and metagenomic potential to comparable levels in healthy individuals.

### Modulation of Host Immunity

Probiotics are known to modulate the host immunity via regulating the innate and adapted cellular and humoral immunity (4, 31, 32). For example, a meta-analysis of clinical trials of *Lactobacillus acidopilus* and *L. plantarum* found that these species could improve host immunity by regulating the secretion of pro-inflammatory and anti-inflammatory cytokines (32, 33). Thus, it is possible that the probiotic administration employed similar mechanisms to exert immunomodulatory effects to patients contracted COVID-19.

Normally, the host immunoregulation is realized by dynamic interactions between immune effector cells (e.g., dendritic cells, macrophages, and lymphocytes) and cytokines (34). Omics tools have identified probiotics-originated immunomodulatory genes and compounds (35); particularly, genes relating to the quorum-sensing system, bacteriocin biosynthesis pathways, stress responses; and compounds such as vitamins and peptides have been found to regulate cytokine production (35). It is worth noting that the clinical outcomes of probiotics-directed immunoregulatory effects are often dependent on multiple factors in the treatment protocol, including the bacterial strain, dose of application, and treatment period (36). Since only limited clinical data are available, it is yet to investigate to what extent probiotics could help maintain gut immune homeostasis in COVID-19 patients.

### Modulation of the Gut-Lung Axis

Coronaviruses are divided into four genera: α, β, γ, and δ (37). Six types of human coronaviruses were discovered before 2019, including two α-coronaviruses, HcoV-229E and HcoV-NL63, and four β-coronaviruses, HcoV-HKU1, HcoV-OC43, severe acute respiratory syndrome coronavirus (SARS-CoV), and Middle East respiratory syndrome coronavirus (MERS-CoV). Four of them, HcoV-229E, HcoV-NL63, HcoV-HKU1, and HcoV-OC43, are usually causing mild upper respiratory tract infections (37), and they rarely invade the lower respiratory tract. In contrast, SARS-CoV and MERS-CoV are highly homologous with SARS-CoV-2, which can spread to the lungs and cause lower respiratory tract infections and severe respiratory syndrome in humans (38, 39). The causative agent of the COVID-19 pandemic, SARS-CoV-2, is a β-coronavirus. It is the seventh known coronavirus that can infect humans. COVID-19 is a lower respiratory tract infection that readily spreads downward and triggers deadly levels of lung inflammation (39). The gastrointestinal tract hosts trillions of resident microorganisms, and an unstable gut microbial community can trigger an onset and/or modulate the outcome of respiratory tract infections (24). Several recent meta-analyses of prebiotic and probiotic studies have reached the same conclusion that ingesting probiotics could effectively prevent respiratory infections and reduce the number of acute respiratory infections without side effects (40–42).

Recently, the gut-lung axis concept has been established, implicating bidirectional communications and mutual dependence between these two body compartments and that alterations of the gut microbial communities may have a profound effect on lung function and disease. Experimental evidence also supports that mucosal immunity is common to different body sites. Both dendritic cells present in the gut and macrophages located in the respiratory tract can collect and present antigens to lymphocytes in nearby lymphoid tissues. Locally activated cells would then further modulate host systemic immunity by circulating through the lymphatic system. Less evidence exists showing direct transfer of microorganisms between body sites, but the transmission of intestinal bacteria to the lungs by inhaling vomit or esophageal reflux has been reported (43). In that case, the transferred microbes would be recognized by immune cells after reaching the distant body compartment, and cytokines would be released in response. Thus, the immune and inflammatory state of the gastrointestinal tract could affect other parts of the body, including the lungs (31, 43). A clinical study concluded that co-administration of oral probiotic during treatment of COVID-19 had eight-fold lower risk of developing respiratory failure compared with the control subjects (41). The fact that the association of gut dysbiosis with an increased mortality in respiratory infections and severity in COVID-19-related symptoms as a result of regulatory dysfunction in inflammatory and/or anti-inflammatory mechanisms in the lungs and in the gut suggests a close connection and vital cross-talks between the two mucosal compartments (44). Further longitudinal studies are needed to clarify the role of the microbiota and gut-lung cross-talks in respiratory diseases, paving the way for future application of specific probiotic strains in managing COVID-19 (18, 45, 46).

## Discussion

Changes in the gut microbiota and immunity have long been used as outcome parameters in RCTs for measuring the clinical efficacy of probiotic adjunctive treatment (47). However, the clinical outcomes described in RCTs for assessing the probiotic functions are still somewhat controversial, and the results obtained from RCTs should be cautiously interpreted because of some common limitations. For example, some RCTs were set up with a relatively small population size, hindering from obtaining a real picture of clinical efficacy and understanding of the mechanisms of the probiotic actions (22). Another plausible reason could be that the original claimed functions of the probiotics appeared to be affected by the manufacturing process (48).

Thus, in future, significantly more high-quality RCTs are necessary to achieve precise understanding of the clinical functions of probiotics as adjunctive treatments for various diseases, particularly in novel diseases like COVID-19. Proper analysis and cautious interpretation from large-scale RCTs that are soon finished will certainly help clarify the role and clinical effects of probiotics application in such cases. Moreover, stringent controls and standards should be followed in manufacturing probiotic products to ensure the product quality and genetic stability of the cells. Last but not least, the success in using probiotics as adjunctive treatment for COVID-19 has expanded the scope of application of probiotics, which would further deepen our knowledge of the physiological function of probiotics in modulating the gut ecosystem and its interactions with the respiratory system.

## Author Contributions

WZ conceived the article and wrote the first draft of the article. JP, MZ, and GY refined the structure of the article and updated the content of the article. L-YK modified the manuscript. All authors approved the final version of the manuscript.

## Conflict of Interest

The authors declare that the research was conducted in the absence of any commercial or financial relationships that could be construed as a potential conflict of interest.

## References

[1] Li G Fan Y Lai Y Han T Li Z Zhou P. Coronavirus infections and immune responses. J Med Virol. (2020) 92:424–32. 10.1002/jmv.25685 31981224 PMC7166547

[2] Chen N Zhou M Dong X Qu J Gong F Han Y. Epidemiological and clinical characteristics of 99 cases of 2019 novel coronavirus pneumonia in Wuhan, China: a descriptive study. Lancet. (2020) 395:507–13. 10.1016/S0140-6736(20)30211-7 32007143 PMC7135076

[3] Chan JF Yuan S Kok KH To KK Chu H Yang J. A familial cluster of pneumonia associated with the 2019 novel coronavirus indicating person-to-person transmission: a study of a family cluster. Lancet. (2020) 395:514–23. 10.1016/S0140-6736(20)30154-9 31986261 PMC7159286

[4] Sundararaman A Ray M Ravindra PV Halami PM. Role of probiotics to combat viral infections with emphasis on COVID-19. Appl Microbiol Biotechnol. (2020) 104:8089–104. 10.1007/s00253-020-10832-4 32813065 PMC7434852

[5] Xu XW Wu XX Jiang XG Xu KJ Ying LJ Ma CL. Clinical findings in a group of patients infected with the 2019 novel coronavirus (SARS-Cov-2) outside of Wuhan, China: retrospective case series. BMJ. (2020) 368:m606. 10.1136/bmj.m606 32075786 PMC7224340

[6] Lee IC Huo TI Huang YH. Gastrointestinal and liver manifestations in patients with COVID-19. J Chin Med Assoc. (2020) 83:521–3. 10.1097/JCMA.0000000000000319 32243269 PMC7176263

[7] Holshue ML DeBolt C Lindquist S Lofy KH Wiesman J Bruce H. First case of 2019 novel coronavirus in the United States. New Engl J Med. (2020) 382:929–36. 10.1056/NEJMoa2001191 32004427 PMC7092802

[8] Chen Y Gu S Chen Y Lu H Shi D Guo J. Six-month follow-up of gut microbiota richness in patients with COVID-19. Gut. (2021) 0:1–3. 10.1136/gutjnl-2021-324090 33833065 PMC8666823

[9] Gu S Chen Y Wu Z Chen Y Gao H Lv L. Alterations of the gut microbiota in patients with coronavirus disease 2019 or H1N1 influenza. Clin Infect Dis. (2020) 71:2669–78. 10.1093/cid/ciaa709 32497191 PMC7314193

[10] Yeoh YK Zuo T Lui GC Zhang F Liu Q Li AY. Gut microbiota composition reflects disease severity and dysfunctional immune responses in patients with COVID-19. Gut. (2021) 70:698–706. 10.1136/gutjnl-2020-323020 33431578 PMC7804842

[11] Lahti L Salojärvi J Salonen A Scheffer M de Vos WM. Tipping elements in the human intestinal ecosystem. Nat Commun. (2014) 5:4344. 10.1038/ncomms5344 25003530 PMC4102116

[12] Dickson RP Singer BH Newstead MW Falkowski NR Erb-Downward JR Standiford TJ. Enrichment of the lung microbiome with gut bacteria in sepsis and the acute respiratory distress syndrome. Nat Microbiol. (2016) 1:16113. 10.1038/nmicrobiol.2016.113 27670109 PMC5076472

[13] Zhang Q Ran X He Y Ai Q Shi Y. Acetate downregulates the activation of NLRP3 inflammasomes and attenuates lung injury in neonatal mice with bronchopulmonary dysplasia. Front Pediatr. (2020) 8:595157. 10.3389/fped.2020.595157 33614540 PMC7889800

[14] Hill C Guarner F Reid G Gibson GR Merenstein DJ Pot B. Expert consensus document. The International scientific association for probiotics and prebiotics consensus statement on the scope and appropriate use of the term probiotic. Nat Rev Gastroenterol Hepatol. (2014) 11:506–14. 10.1038/nrgastro.2014.66 24912386

[15] Baud D Dimopoulou Agri V Gibson GR Reid G Giannoni E. Using probiotics to flatten the curve of coronavirus disease COVID-2019 pandemic. Front Public Health. (2020) 8:186. 10.3389/fpubh.2020.00186 32574290 PMC7227397

[16] Xu K Cai H Shen Y Ni Q Chen Y Hu S. [Management of corona virus disease-19 (COVID-19): the Zhejiang experience]. Zhejiang Da Xue Xue Bao Yi Xue Ban. (2020) 49:147–57. 10.3785/j.issn.1008-9292.2020.02.02 32391658 PMC8800711

[17] Chunyan W Qian X Zhan C Dengdeng P Ying Z Sheng W. The volatile and heterogenous gut microbiota shifts of COVID-19 patients over the course of a probiotics-assisted therapy. Res Square. (2021) 0:1–16. 10.21203/rs.3.rs-72753/v1 PMC8713143 34962356

[18] Baindara P Chakraborty R Holliday ZM Mandal SM Schrum AG. Oral probiotics in coronavirus disease 2019: connecting the gut-lung axis to viral pathogenesis, inflammation, secondary infection and clinical trials. New Microb New Infect. (2021) 40:100837. 10.1016/j.nmni.2021.100837 33425362 PMC7785423

[19] Hu J Zhang L Lin W Tang W Chan FKL Ng SC. Review article: probiotics, prebiotics and dietary approaches during COVID-19 pandemic. Trends Food Sci Technol. (2021) 108:187–96. 10.1016/j.tifs.2020.12.009 33519087 PMC7833886

[20] Zhao L. Rutgers Researcher Invents Microbiota Formula to Help High Risk Patients Fight COVID-19. New Brunswick, NJ: Rutgers The State University of New Jersey (2021).

[21] Suvorov A. Gut microbiota, probiotics, human health. Biosci Microb Food Health. (2013) 32:81–91. 10.12938/bmfh.32.81 24936366 PMC4034364

[22] Ding RX Goh WR Wu RN Yue XQ Luo X Khine WWT. Revisit gut microbiota and its impact on human health and disease. J Food Drug Anal. (2019) 27:623–31. 10.1016/j.jfda.2018.12.012 31324279 PMC9307029

[23] Ferreira C Viana SD Reis F. Gut microbiota dysbiosis-immune hyperresponse-inflammation triad in coronavirus disease 2019 (COVID-19): impact of pharmacological and nutraceutical approaches. Microorganisms. (2020) 8:1514. 10.3390/microorganisms8101514 33019592 PMC7601735

[24] Walton GE Gibson GR Hunter KA. Mechanisms linking the human gut microbiome to prophylactic and treatment strategies for COVID-19. Br J Nutr. (2020) 0:1–9. 10.1017/S0007114520003980 33032673 PMC7684010

[25] Hiippala K Jouhten H Ronkainen A Hartikainen A Kainulainen V Jalanka J. The potential of gut commensals in reinforcing intestinal barrier function and alleviating inflammation. Nutrients. (2018) 10:988. 10.3390/nu10080988 30060606 PMC6116138

[26] Giannoni E Baud D Agri VD Gibson GR Reid G. Probiotics and COVID-19. Lancet Gastroenterol Hepatol. (2020) 5:720–1. 10.1016/S2468-1253(20)30195-3 PMC7357984 32673603

[27] Din AU Mazhar M Waseem M Ahmad W Bibi A Hassan A. SARS-CoV-2 microbiome dysbiosis linked disorders and possible probiotics role. Biomed Pharmacother. (2021) 133:110947. 10.1016/j.biopha.2020.110947 33197765 PMC7657099

[28] Yu LC Wang JT Wei SC Ni YH. Host-microbial interactions and regulation of intestinal epithelial barrier function: from physiology to pathology. World J Gastrointest Pathophysiol. (2012) 3:27–43. 10.4291/wjgp.v3.i1.27 22368784 PMC3284523

[29] Yang Q Liang Q Balakrishnan B Belobrajdic DP Feng QJ Zhang W. Role of dietary nutrients in the modulation of gut microbiota: a narrative review. Nutrients. (2020) 12:381. 10.3390/nu12020381 32023943 PMC7071260

[30] Zuo T Liu Q Zhang F Lui GC Tso EY Yeoh YK. Depicting SARS-CoV-2 faecal viral activity in association with gut microbiota composition in patients with COVID-19. Gut. (2021) 70:276–84. 10.1136/gutjnl-2020-322294 32690600 PMC7385744

[31] Stavropoulou E Bezirtzoglou E. Probiotics as a weapon in the fight against COVID-19. Front Nutr. (2020) 7:614986. 10.3389/fnut.2020.614986 33385008 PMC7769760

[32] Zhao W Peng C Sakandar HA Kwok LY Zhang W. Meta-analysis: randomized trials of *Lactobacillus plantarum* on immune regulation over the last decades. Front Immunol. (2021) 12:643420. 10.3389/fimmu.2021.643420 33828554 PMC8019694

[33] Zhao W Liu Y Kwok L-Y Cai T Zhang W. The immune regulatory role of *Lactobacillus acidophilus*: an updated meta-analysis of randomized controlled trials. Food Biosci. (2020) 36:100656. 10.1016/j.fbio.2020.100656

[34] Ya T Zhang Q Chu F Merritt J Bilige M Sun T. Immunological evaluation of *Lactobacillus casei* Zhang: a newly isolated strain from koumiss in inner Mongolia, China. BMC Immunol. (2008) 9:68. 10.1186/1471-2172-9-68 19019236 PMC2596084

[35] Yan F Polk DB. Probiotics and immune health. Curr Opin Gastroenterol. (2011) 27:496–501. 10.1097/MOG.0b013e32834baa4d 21897224 PMC4006993

[36] Kang HJ Im SH. Probiotics as an immune modulator. J Nutr Sci Vitaminol. (2015) 61:S103–5. 10.3177/jnsv.61.S103 26598815

[37] Chan JF Lau SK To KK Cheng VC Woo PC Yuen KY. Middle East respiratory syndrome coronavirus: another zoonotic betacoronavirus causing SARS-like disease. Clin Microbiol Rev. (2015) 28:465–522. 10.1128/CMR.00102-14 25810418 PMC4402954

[38] Gaunt ER Hardie A Claas EC Simmonds P Templeton KE. Epidemiology and clinical presentations of the four human coronaviruses 229E, HKU1, NL63, and OC43 detected over 3 years using a novel multiplex real-time PCR method. J Clin Microbiol. (2010) 48:2940–7. 10.1128/JCM.00636-10 20554810 PMC2916580

[39] Duarte R Furtado I Sousa L Carvalho CFA. The 2019 novel coronavirus (2019-nCoV): novel virus, old challenges. Acta Med Port. (2020) 33:155. 10.20344/amp.13547 32023427

[40] Wang Y Li X Ge T Xiao Y Liao Y Cui Y. Probiotics for prevention and treatment of respiratory tract infections in children: a systematic review and meta-analysis of randomized controlled trials. Medicine. (2016) 95:e4509. 10.1097/MD.0000000000004509 27495104 PMC4979858

[41] d'Ettorre G Ceccarelli G Marazzato M Campagna G Pinacchio C Alessandri F. Challenges in the management of SARS-CoV2 infection: the role of oral bacteriotherapy as complementary therapeutic strategy to avoid the progression of COVID-19. Front Med. (2020) 7:389. 10.3389/fmed.2020.00389 32733907 PMC7358304

[42] Hao Q Dong BR Wu T. Probiotics for preventing acute upper respiratory tract infections. Cochr Database Syst Rev. (2015) 2:CD006895. 10.1002/14651858.CD006895.pub3 25927096

[43] Marsland BJ Trompette A Gollwitzer ES. The gut-lung axis in respiratory disease. Ann Am Thor Soc. (2015) 12(Suppl. 2):S150–6. 10.1513/AnnalsATS.201503-133AW 26595731

[44] de Oliveira GLV Oliveira CNS Pinzan CF de Salis LVV Cardoso CRB. Microbiota modulation of the gut-lung axis in COVID-19. Front Immunol. (2021) 12:635471. 10.3389/fimmu.2021.635471 33717181 PMC7945592

[45] Akour A. Probiotics and COVID-19: is there any link? Lett Appl Microbiol. (2020) 71:229–34. 10.1111/lam.13334 32495940 PMC7300613

[46] Mak JWY Chan FKL Ng SC. Probiotics and COVID-19: one size does not fit all. Lancet Gastroenterol Hepatol. (2020) 5:644–45. 10.1016/S2468-1253(20)30122-9 32339473 PMC7182525

[47] Dickerson F Adamos M Katsafanas E Khushalani S Origoni A Savage C. Adjunctive probiotic microorganisms to prevent rehospitalization in patients with acute mania: a randomized controlled trial. Bipolar Disord. (2018) 20:614–21. 10.1111/bdi.12652 29693757

[48] Sybesma W Molenaar D van IJcken W Venema K Kort R. Genome instability in *Lactobacillus rhamnosus* GG. Appl Environ Microbiol. (2013) 79:2233–9. 10.1128/AEM.03566-12 23354703 PMC3623246

